# Epidemiology, clinical presentation and treatment outcomes in patients with COVID-19 in an ambulatory setting: a cross sectional study during the massive SARS-CoV-2 wave in India

**DOI:** 10.6026/97320630019939

**Published:** 2023-09-30

**Authors:** Sushil Sharma, Rakesh Upparakadiyala, Santenna Chenchula, Madhavrao Chavan, Gaurav Rangari, Arup Kumar Misra

**Affiliations:** 1 Department of Pharmacology, All India Institute of Medical Sciences, Mangalagiri, India; 2 Department of General Medicine, All India Institute of Medical Sciences, Mangalagiri, 522503, India

**Keywords:** COVID-19, SARS-CoV-2, clinical characteristics, hospitalization, medical comorbidities, mortality

## Abstract

The COVID-19 pandemic, caused by SARS-CoV-2, has profoundly affected developing countries like India. This retrospective cross-sectional analysis investigated epidemiological, clinical characteristics, treatment strategies, and outcomes for hospitalized COVID-19 patients during the Massive SARS-CoV-2 Wave in India. Among 233 patients, the median age was 47.33 years, mostly male. Hospital stays averaged 8.4 days. Common symptoms include fever (88.41%), dry cough (56.2%), myalgia (44.20%), and shortness of breath (22.8%). The most common comorbidities were diabetes mellitus (52%) and hypertension (47.2%). Elevated biomarkers include D-dimer (24.4%), CRP (32.1%), ferritin (26.60%), and others. Prescription analysis revealed that antibiotics (42.6%), Antivirals (37%), anthelmintics (20.30%), vitamins and nutritional supplements (20.71%) and glucocorticoids (12.8%) were the most commonly prescribed. Oxygen therapy was needed by 19.31% of patients in the moderate and severe categories within 24 hours of admission. The mortality rate was 8.58%. The surge led to increased hospitalizations and mortality, particularly among young adults. Diabetes and hypertension were correlated with mortality. Irregular use of drugs lacking evidence, like antibiotics and anthelmintics, vitamins and nutritional supplements, was observed in COVID-19 management. This study underscores the impact of the pandemic in India and highlights the need for evidence-based treatments.

## Background:

Corona Virus Disease-2019 (COVID-19) is a current global pandemic, caused by severe acute respiratory syndrome coronavirus-2 (SARS-CoV-2), a single-stranded positive-sense RNA (ssRNA) virus classified in the group of the coronaviridae family. [1]. Till February 2023, cases of COVID-19 have surpassed 650 million and 6.5 million deaths across the world [2]. SARS-CoV-2 is diagnosed by reverse transcription-polymerase chain reaction (RT-PCR) and is the gold standard method [3]. Most commonly, SARS-CoV-2 transmit from one person to another through respiratory particles due to close-range contact or by contact with a contaminated surface [4, 5]. Very common clinical symptoms include high-grade fever, dry cough, sore throat, body ache, headache, fatigue, loss of taste and smell, abdominal pain, and diarrhoea [6]. In severe form, it may lead to dyspnea, pneumonia, acute respiratory distress syndrome (ARDS) due to cytokine release syndrome, multiorgan dysfunction and death [6, 7]. The most common comorbidities associated with the increased severity of COVID-19 were hypertension, diabetes, obesity, and cardiovascular diseases [8]. Several variants of SARS-CoV-2 have emerged that are notable because of their potential for increased transmission [9]. In addition to various vaccines, several pharmacological agents have been repurposed against coronavirus infection including, hydroxychloroquine, remdesivir, favipiravir, famotidine, glucocorticoids, immune modulators (IL-6 inhibitors, JAK-STAT Inhibitors) and Monoclonal antibodies, etc., based on evidence from the *in vitro* and *in vivo* studies data [10, 11,12]. Understanding of COVID-19 is still evolving, and information regarding hospitalized patients with COVID-19 is very essential for future readiness to fight against the pandemic. Unfortunately, there is a dearth of published data on COVID-19 pathophysiology, disease course, baseline characteristics, risk factors, effective treatment strategies and outcomes in hospitalized COVID-19 patients in India. Therefore, it is of interest to describe the demographic data, baseline comorbidities, presenting clinical tests, pharmaco-therapeutic agents and outcomes of hospitalized patients with COVID-19 from a tertiary care academic healthcare institute, India.

## Methodology:

The present retrospective cross-sectional study was conducted at a tertiary care national institute All India Institute of medical sciences (AIIMS), Mangalagiri, India. Medical records of all the hospitalized patients with confirmed RT-PCR testing of the nasopharyngeal and oropharyngeal swabs by a positive result on reverse transcriptase polymerase chain reaction (RT-PCR) is collected. Data collected includes patient demographic information, comorbidities, signs and symptoms, laboratory tests, electrocardiogram results, radiographic examination results (Chest X-ray or CT-Chest), inpatient medications, treatments (including invasive mechanical ventilation), and outcomes (including length of stay, discharge and mortality). The following data were retrieved from the patient records by manual as well as electronic data recordkeeping system: demographic information, comorbidities, signs and symptoms, and any available investigations (complete blood count, serum ferritin, D dimer, CRP, ESR, LDH, liver and kidney function tests, electrocardiogram (ECG), chest X-ray or CT-Chest *etc.*), inpatient medications, treatments (including respiratory support by oxygen therapy by facemask, a high-flow nasal cannula(HFNC), non-invasive or invasive mechanical ventilation), and outcomes (including length of stay, discharge and mortality). We followed the WHO and revised Indian Council of medical research (ICMR) guidelines (released on 31.03.2021)" for standardized definitions of the clinical syndrome of COVID-19 [13]. Collected data were reviewed by two researchers independently to double-check the collected data. This was done to improve the quality of data collected and entered into a password-protected computer Excel database.

## Statistical Analysis:

Statistical analysis was performed in STATA version 21 (StataCorp LP, TX, USA). All the continuous variables data were presented as Mean ± Standard Deviation (SD) median (interquartile range, IQR) and categorical data were presented as absolute numbers and percentages. Non-parametric tests were used for all analyses, and clinical characteristics were compared between survivors and non-survivors. For all the statistical analyses, a p-value < 0.05 will be considered statistically significant.

## Results:

A total of 233 COVID-19 patient records were analyzed in the present study. The median age of study subjects was 47.33±15.1 years and the majority were males (n=152; 65.24%). The median (IQR) duration of hospitalization was 8.4 days. At the time of presentation, patients were categorized according to the WHO-ICMR guidelines for standardized definitions of the clinical syndrome of COVID-19 into mild or uncomplicated (31.8%), moderate (54.6%) and severe (13.8 %) categories. At the time of admission oxyhemoglobin saturation (SpO2) at room air was recorded in COVID-19 patients of the present study and was as follows:< 90 mm Hg in 15.46%(n=36), 90-94 mm Hg in 66.1(n=154), and >94 mm Hg in 18.46% (n=43) patients respectively. Fever (88.41%), dry cough (56.22%) and wet cough (43.77%), myalgia (44.20%), shortness of breath (22.8%), rhinitis (21.5%), sore throat (20.60%), fatigue (20.6%), loss of smell and taste (16.74%), weakness (12%), diarrhoea (8.5%) and abdominal pain (0.43%) were the most common symptom. A total of 125 (53.64%) patients have been suffering from concomitant comorbidity. Diabetes mellitus (n=65;52%), and hypertension (n=59; 47.2%) were the most common comorbid illness. In addition, hypothyroidism (10.4%), coronary artery disease (8.1%), chronic kidney disease (4%), asthma (2.4%), heart failure (1.6%), Parkinson's disease (1.6%), and arthritis (1.6%) were present in patients with COVID-19. All the baseline demographic data and clinical characteristics and comorbidities were summarized in the Table 1.

Analysis of Laboratory and radiological investigations was done. Elevated levels of inflammatory biomarkers such as serum D-dimer (24.46%), CRP (32.18%), ferritin (26.60%), ESR (4.3%), LDH (8.6%), blood urea nitrogen (17.2%), creatinine (9.01%), and random blood sugar (RBS)>140 mg/dl (24.03%) were found in the hospitalized patients with COVID-19. Abnormal electrocardiogram findings have been seen in 20(8.6%) patients and sinus tachycardia (6.4%) was the most common abnormality followed by ischemic changes (2.14%). Anemia was diagnosed in only 5(0.42%) patients. Chest X-ray report was available only in 21(9.01%) patients and a total of 18 (7.8%) patients have shown the characteristics of COVID-19, bilateral ground glass opacities in lower lobes of lungs. HRCT Chest report was available in only 4 (1.71%) patients with a significantly higher score with consolidation, and fibrosis changes. All the Laboratory and radiological investigations data are summarized in Table 2.

A total of 3208 drugs were prescribed. Among them, 2612 (81.42) were non-antimicrobials and 596 (18.57%) were antimicrobials. Antimicrobial agents include a total of 37.08% antivirals, 42.62% of antibiotics, 20.30% antihelminthic,2.75% antifungals, 2.36% of other antimicrobials such as metronidazole and ofloxacin. Among antivirals majority were remdesivir 218(98.64 %) followed by favipiravir (3;1.4%). Among antibiotics, 43.70% were tetracycline group, 39.76% were beta-lactams with the majority as a combination of a beta-lactam plus beta-lactamase (66.33%). However, only one patient (0.16%) had received the antimalarial agent hydroxychloroquine.

Among 2612 (81.42%) the non-antimicrobials majority were vitamins and protein supplements (541, 20.71) followed by glucocorticoids (12.82%) and Antitussives (9.8%), non-steroidal anti-inflammatory drugs (9.35%). Other non-antimicrobials include 6.9% of PPIs, 6.4% of antihistamines, 6.3% of anticoagulants, 88.62% of leukotriene receptor blockers, 3.9% of insulin, 2.9% oral antidiabetic agents, 2.4% of antihypertensives, 1.84% of oral antiseptics, 1.45% of bronchodilators, 0.95% of probiotics, 0.57% of thyroid hormone supplements, 0.34% of HMG-CoA reductase inhibitors or statins, 0.11% of Janus kinase inhibitors and 7.6 % of other agents. A total of 45 (19.31%) patients from the severe and moderate categories have been treated with oxygen within 24 h of hospital admission.

Among all the hospitalized patients, twenty patients (8.58%) from the severe category died during the period of hospital stay. The mean age among non-survival was (52.90 ± 15.751 years) and the baseline SpO2 was lower than survivors at presentation. The average duration of hospital stay was similar between survivors and non-survivors. A total of 24 comorbidities were found in non-survivors, the majority had diabetes mellitus (37.5%), hypertension (33.33%), Heart failure (8.33%), hypothyroidism (8.33%), chronic kidney disease (8.33%) and Parkinson's disease (4.16%). A total of 313 drugs were prescribed among non-survivors. Among the total drugs prescribed, 79 (25.24) were antimicrobials and 234 (74.76) were non-antimicrobials. Among antimicrobials, 26 (8.31) were antivirals followed by 20(25.31%) were antibiotics and 16(5.11) were anthelmintic agents. A total of 16.66% of glucocorticoids and 15.81 % of vitamins and protein nutritional supplements were the most commonly prescribed non-antimicrobial agents. Table 3 presents the Prescription patterns of drugs for hospitalized patients with COVID-19.

## Discussion:

To the best of our knowledge, this study represents the first large case series of sequentially hospitalized patients with confirmed COVID-19 from South India. After the five months gap from the first wave in India, on February 11, 2021, the second wave of COVID-19 started, which has been disastrous, with 0.2 million cases per day in mid-April 2021, which is quadruple that of the first wave peak [14,15]. The number of cases and deaths has risen drastically in India [15]. It was perplexing and caused devastation around the world that the number of COVID-19 cases has increased so quickly after such a small gap from the first wave. In May 2021, the second wave spread to rural areas of India too [15,16]. Several states across India have been affected severely by the second wave [16]. On April 10, 2021, India has been in third place based on the cases discovered by the USA and Brazil [16]. The emergence of mutated COVID-19 virus variants including B.1.617.2(Delta variant) has been associated with the surge in cases during the second wave in India [16].

Demographic and baseline clinical characteristics of the cases of COVID-19 in the present study were similar to the population from other parts of India [17]. The median age (48 yrs.) and the ratio of the male sex of hospitalized COVID-19 patients during the Delta wave are in line with other studies published in India during the Delta wave of COVID-19 [18,19, 20]. However, in the present study, the average age of non-survivors was higher than survivors. majority of hospitalized patients (111; 43.35%) had moderate to severe COVID-19 was higher among the population aged >40 years with a higher proportion of patients in the younger age group intervals of 18-40 yrs. Fever, cough, myalgia and shortness of breath were the most commonly reported symptoms similar to other studies in India [18,19,20]. High-grade fever (>103°F) was found in 10.19% of patients in the present study. Dry cough (56.22%) was the most common cough followed by wet cough (43.77%). However, during the first wave of COVID-19 also fever, cough and shortness of breath were the predominant symptoms. Concomitant medical comorbidities were significantly associated with increased severity and hospitalization among patients with COVID-19 [21]. Among the 125 patients with comorbidities, a total of 93 (74.4%) patients have at least one comorbidity and 32 (25.6%) have more than one comorbidity. Diabetes mellitus and hypertension were the most common comorbidities among both non-survivors and survivors in the present study which is similar to studies published in other parts of India [18, 19,20].

Laboratory parameters CRP, ferritin, ESR, LDH and D dimer are essential to estimate the severity of COVID-19 [22]. Laboratory evidence with these abnormal laboratory parameters has been associated with critical and fatal illnesses and in response to T cell immunotherapy to cytokine storm and sepsis [22]. Elevated levels of inflammatory markers such as CRP, ferritin, ESR, LDH and D dimer were found in the present study. Ferritin is the most commonly elevated inflammatory biomarker in COVID-19 and hyper ferritinemia is associated with increased severity of COVID-19 leading to cytokine release syndrome [22]. Elevated LDH levels are a marker of severe lung injury leading to ARDS and increased mortality [23,24]. In the present study, the majority of moderate to severe patients in the present study has shown an increased level of LDH. Increased levels of serum creatinine and blood urea nitrogen are also associated with acute kidney injury (AKI) with severe COVID-19 in hospitalized patients with corona disease [25]. A total of 9.01% increased serum creatinine, and 17.2% blood urea nitrogen have been found in the present study population. An abnormal increase in RBC from the normal range (>140 mg/dl) was seen in the study population. Previous studies have shown that severe COVID-19 is associated with new-onset diabetes [26]. Several hypothetical mechanisms were proposed for hyperglycemia associated with COVID-19 includes, including increased use of corticosteroids, previously undiagnosed diabetes, stress hyperglycemia and direct or indirect effects of SARS-CoV-2) on the β-cell [26]. Studies from other regions of India also have shown similar findings of hyperglycemia [16,20]. Among the 21 chest X-ray reports, only 18 (7.8%) patients had chest X-ray abnormalities, and the most common abnormality was found as bilateral ground glass opacities in the lower lobes of the lungs. The HRCT Chest report was abnormal in 4 (1.71%) available reports with significant consolidation and fibrosis changes. Both X-ray and HRCT reports were similar to other studies in India [18,20]. In the present study, the most common abnormal electrocardiogram findings include sinus tachycardia and ischemic changes.

In the current study, a total of 81.42% of prescribed drugs were non-antimicrobials and 18.57% were antimicrobials. Among the antimicrobials majority were antivirals (37.08%) with 98.64% of remdesivir and only 1.4% of favipiravir. However, a meta-analysis of remdesivir clinical trials has shown that it doesn't decrease any mortality in hospitalized patients with moderate to severe COVID-19 [27]. Among tetracyclines group antibiotics, the most commonly prescribed was doxycyclin. Amoxycillin, cefalosporins, piperacillin, meropenem and faropenem were the most commonly prescribed beta-lactam antibiotics. Azithromycin was the most commonly prescribed macrolide and ivermectin was the most commonly prescribed anthelmintic agent. Previous studies have shown that doxycycline and ivermectin only help in the early course of the disease in decreasing the mild-to-moderate COVID-19 infection progression to more severe disease, but no evidence of efficacy among hospitalized severe COVID-19 patients [28, 29]. Studies have shown that the Prevalence of bacterial coinfection in COVID-19 is low, yet a high consumption of antimicrobials was seen in COVID-19 patients [30,31]. Studies have shown that, in the year 2019, Antimicrobial resistance (AMR) was associated with the death of more than 1.2 million people [32]. Therefore, unjudicial use of antibiotics should be stopped for the management of COVID-19 disease. Vitamin & nutritional supplements (20.71) followed by corticosteroids (12.82) and antitussives were the most commonly prescribed nonantimicrobial agents. most commonly prescribed vitamins & Nutritional supplements include, vitamin C, vitamin D plus calcium and protein supplements. Among glucocorticoids, 39.10% methylprednisolone followed by 30.74% dexamethasone and 30.14% budesonide. The most commonly prescribed vitamin supplements include Vitamin C (ascorbic acid), and vitamin D (Cholecalciferol), followed by micronutrients zinc and protein supplements were the most commonly prescribed vitamin & nutritional supplements. They are considered immunity-boosting agents for the prevention and treatment of viral infections, however, there is a lack of evidence on their efficacy for the treatment or reduce the severity of COVID-19 infections [33,34]. Evidence from the randomized trials supports the use of glucocorticoids for moderate to severe COVID-19 [35,36]. However high-dose glucocorticoids use among COVID-19 patients was significantly associated with an increased risk of mucormycosis [37]. In the present study also, three patients were diagnosed to have mucormycosis and were treated with systemic antifungal antibiotic drugs. The most commonly prescribed NSAID was paracetamol (93.36%), dextromethorphan for dry cough followed by ambroxol in combination with guaifenesin was the most commonly prescribed mucolytic agent for wet cough. Levosalbutamol and acebrophylin were the most commonly prescribed bronchodilators. Pantoprazole was the most commonly prescribed PPIs (95%). Baricitinib was the most commonly prescribed Janus kinase inhibitor. Povidone iodine was the most commonly prescribed oral antiseptic agent for mouth gargling. A total of 19.31% of patients have required oxygenation in the present study including all non-survivors. Based on the evidence from the clinical trial studies of the first wave, the antimalarial agent hydroxychloroquine use was significantly decreased in the present study centre as well as in other parts of India and across the world during the Delta wave [38]. In the current era of antimicrobial resistance across the world, irrational use of antibiotics for the treatment of antivirals, fuels the emergence of multidrug resistant bacteria [39,40, 41,43]. Currently highly infectious omicron variants and novel viral infections like Mpox, stake holders and policy makers must take significant action regarding irrational use of antimicrobial agents across the world [44,45].

Statistical modelling studies have shown that the average case fatality ratio in the first wave was lower than in the Delta wave in all of the southern Indian states [46]. During the first wave of COVID-19 mortality was higher among males, and aged >60 years [18,20].in the present study, the mortality was 8.58 per cent. The median age among non-survivals was higher than survivors (53 years) and the baseline SpO2 was lower than survivors at presentation. Most common comorbidities associated with increased mortality and morbidity among non-survivors includes diabetes mellitus (37.5%), hypertension (33.33%), heart failure (8.33%), hypothyroidism (8.33%), and chronic kidney disease (8.33%). The average duration of hospital stay was similar between survivors and non-survivors. The most common reason for the death of the patients was acute respiratory distress syndrome with severe COVID-19. Among non-survivors, higher baseline serum LDH, D-dimer, CRP, and ferritin levels were found. Shortness of breath (35%) was the most common symptom at the presentation and the RBS levels were higher than the normal range among all the non-diabetic patients in non-survivors. The present study has some limitations. It is a single-centre study; hence the number of patients was lower. Because it is a retrospective analysis study, data on laboratory parameters and other data were missed for many patients hence it was not collected for many patients. Due to the low event rate of outcome (mortality), a multivariate analysis to identify risk factors could not be performed.

## Conclusion:

The present retrospective study provides detailed clinical characteristics and treatment outcomes of hospitalized patients with confirmed COVID-19 in India during the Delta wave of COVID-19. The majority of patients hospitalized with COVID-19 presented were moderate to severe disease. During the Delta wave, the majority of patients hospitalized with severe COVID-19 were younger adults below 50 years and the majority were males. Mortality was 8.58%. Diabetes mellitus and hypertension were the most common comorbidities among both nonsurvivors and survivors and higher baseline serum LDH, D-dimer, CRP, ferritin and RBS levels among moderate to severe COVID-19 patients. Irrational Use of drugs that lack evidence such as remdesivir and nutritional supplements for the treatment of COVID-19 increase the economic burden and an unjudicial use of antimicrobials, leading to AMR, a major cause of death globally should be stopped.

## Ethical Approval:

The institute's Ethics Committee approval received (IEC: 2021-22/117)

## Funding:

All authors have declared that no financial support was received from any organization for the submitted work.

## Authors'contributions:

All authors meet the ICMJE criteria for authorship. SC, conducted the Patient records search, data extraction and drafted the manuscript, SS, MC, RU, GR, and AKM reviewed and revised the manuscript. All authors have reviewed and approved the final version of the manuscript.

## Data availability:

The datasets generated during and/or analysed during the current study are available from the corresponding author on reasonable request.

## Figures and Tables

**Table 1:** Baseline vitals, presenting characteristics, and comorbidities of the patients hospitalized with covid-19 on the day of hospitalization

**Demographic information**	**Total Number (%)**
Total number of Patients with COVID-19	233
Total number of Patients Died/ Mortality	20 (8.58)
The Median Duration of Hospital stay	8.4 days
**Age (mean)**	47.33±15.056
<18	2(0.86%)
18-40	89(36.48%)
41-60	111(43.35%)
61-80	47(17.60%)
>80	4(1.72%)
**Sex**
Male	152(65.24%)
Female	81(34.76%)
**Oxygen saturation(SPO2)**	Total n (%)
<90	36(15.46)
≥ 90-94	154 (66.1)
≥ 94	43 (18.46)
Received supplemental oxygen at triage	45 (19.31)
Temperature >37.7°C or >99.9° F	206 (88.41)
**Case severity**
Mild or uncomplicated	74 (31.76%)
Moderate	127(54.51%)
severe	32(13.73%)
**Symptoms**	Total n (%)
Wet Cough	102(43.77)
Dry Cough	131(56.22)
Myalgia	103 (44.20)
Shortness of Breath	60 (22.8)
Sore Throat	55 (20.60)
Rhinitis	50 (21.5)
Fatigue	48 (20.6)
Loss of Smell/taste	40 (16.74)
Headache	23(9.87)
DiarrhOea	21 (8.5)
Abdominal Pain	1 (0.43)
**Comorbidities**
Total No. Of patients with comorbidities	125(53.64%)
Patients with at least one co morbidity	93 (74.4%)
Patients with more than one co morbidity	32 (25.6%)
**Cardiovascular disease**
Hypertension	59 (47.2%)
Coronary Artery Disease	10 (8.1%)
Heart failure	2(1.6%)
**Chronic respiratory disease**
Asthma	3(2.4%)
**Endocrine and Metabolic disorders**
Diabetes Mellitus	65 (52%)
Hypothyroidism	13 (10.4%)
**Kidney disease**
Chronic Kidney Disease	5 (4.0%)
**Others**
Parkinson's Disease	2 (1.6%)
Arthritis2 (1.6%)

**Table 2:** Presentation of laboratory results of patients hospitalized with covid-19 on the day of hospitalization

** Parameter**	** Total n (%)**
Laboratory investigations	Total number of patients with Abnormal levels n (%)
X-ray	18(7.8%)
CT Chest	4 (1.71%)
D dimer	57(24.46%)
CRP	70(32.18%)
Serum Ferritin	62 (26.60%)
ESR	10(4.3%)
LDH	20(8.6%)
Serum Creatinine	21(9.01%)
Blood urea nitrogen	40 ((17.2%)
RBS>140 mg/dl	56(24.03%)
Hemoglobin	5(0.42%)
Electrocardiogram (ECG))	20(8.6%)

**Table 3:** Prescription patterns of drugs for hospitalized patients with COVID-19

**Drugs**	**Number of Drugs**
Total drugs	3208
Antimicrobials	596 (18.57)
Antivirals	221 (37.08)
Remdesivir	218(98.64 )
Favipiravir	3(1.4)
Antihelminthics	121 (20.30)
Antibiotics	254(42.61)
Tetracyclines	111 (43.70)
Macrolides	29(11.41)
Beta lactams	101(39.76)
Betalactams with Beta lactamase	67(66.33)
Antifungal agents	7(2.75)
Others	6(2.36)
Non-Antimicrobials	2612 (81.42)
Vitamins & Nutritional supplements	541 (20.71)
Corticosteroids	335(12.82)
Antitussives	256(9.8)
NSAIDs	241(9.3)
Proton pump inhibitors (PPIs)	180(6.9)
Antihistamines	167(6.4)
Anticoagulants	164(6.3)
Leukotrienes + Antihistamines	148(88.62)
Insulin	102(3.9)
Oral antidiabetics	76(2.9)
Antihypertensives	65(2.4)
Oral antiseptics(betadine)	48(1.84)
Bronchodilators	38 (1.45)
Probiotics	25(0.95)
Thyroid hormone(thyroxine)	15(0.57%)
HMG-CoA Reductase Inhibitors or Statins	9(0.34)
Janus kinase inhibitors	3(0.11)
Others	214(8.19)
